# Outbreak.info Research Library: A standardized, searchable platform to discover and explore COVID-19 resources and data

**DOI:** 10.1101/2022.01.20.477133

**Published:** 2022-02-01

**Authors:** Ginger Tsueng, Julia Mullen, Manar Alkuzweny, Marco Cano, Haag Rush Benjamin, Outbreak Emily, Latif Curators, Abdel Alaa, Xinghua Zhou, Zhongchao Qian, Kristian G. Andersen, Chunlei Wu, Andrew I. Su, Karthik Gangavarapu, Laura D. Hughes

## Abstract

To combat the ongoing COVID-19 pandemic, scientists have been conducting research at breakneck speeds, producing over 52,000 peer reviewed articles within the first 12 months. In contrast, a little over 1,000 peer reviewed articles were published within the first 12 months of the SARS-CoV-1 pandemic starting in 2002. In addition to publications, there has also been an upsurge in clinical trials to develop vaccines and treatments, scientific protocols to study SARS-CoV-2, methodology for epidemiological modeling, and datasets spanning molecular studies to social science research. One of the largest challenges has been keeping track of the vast amounts of newly generated disparate data and research that exist in independent repositories. To address this issue, we developed outbreak.info, which provides a standardized, searchable interface of heterogeneous data resources on COVID-19 and SARS-CoV-2. Unifying metadata from 14 data repositories, we have assembled a collection of over 200,000 publications, clinical trials, datasets, protocols, and other resources as of October 2021. We used a rigorous schema to enforce a consistent format across different data sources and resource types, and linked related resources where possible. This enables users to quickly retrieve information across data repositories, regardless of resource type or repository location. Outbreak.info also combines the combined research library with spatiotemporal genomics data on SARS-CoV-2 variants and epidemiological data on COVID-19 cases and deaths. The web interface provides interactive visualizations and reports to explore the unified data and generate hypotheses. In addition to providing a web interface, we also publish the data we have assembled and standardized in a high performance public API and an R package. Finally, we discuss the challenges inherent in combining metadata from scattered and heterogeneous resources and provide recommendations to streamline this process to aid scientific research.

## Introduction

**Figure 1:**
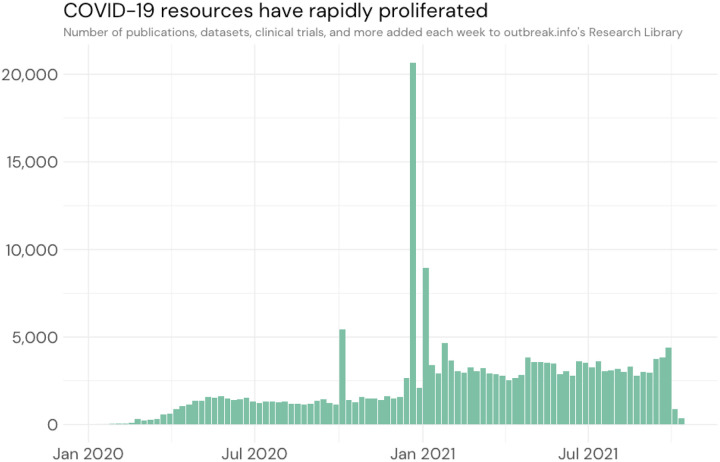
Number of resources in outbreak.info as a function of date.

On the 7th of January 2020, the virus responsible for a series of cases of pneumonia of unknown origin circulating in Wuhan, China since December 2019, was identified as a novel coronavirus, SARS-CoV-2 (World Health Organization, 2020). As the virus quickly spread all over the world, the scientific community began devoting a large amount of time and resources to understand the new virus, disease, and the overall spread of the pandemic. In the course of the proliferation of both the disease and the scientific research, the sheer volume of publications, clinical trials, datasets, protocols and methodology, and more exploded. By April 2020, over 1,000 different resources were being published on a weekly basis (Figure 1). Not only is the volume of research rapidly expanding, but the types of resources are disparate, their locations scattered across the internet, and their metadata described using wildly different standards.

As researchers, public health officials, and concerned communities independently launched personal efforts for improving regional understanding of the epidemiological landscape, so too did the number of sites providing highly localized or specialized information on infection rates, prevention policies, travel restrictions and more (Dong, Du, & Gardner, 2020)(Kaiser, 2020)(Noren, Neff, and Li, 2020) (Morris and citizen scientists, 2020) (James and citizen scientists, 2020) (Pogkas et al., 2020). This initial explosion of individual websites, collections, and resources was followed by individual and community efforts for curating these sites via shared google spreadsheets (Joachimiak et al., 2020) (Skenderi et al., 2020) (Navarro and Capdarest-Arest, 2020). While the sheets provided a list of urls linking to the various COVID-19 sites, most do not include sufficient metadata for meaningful searches with the exception of Navarro and Capdarest-Arest. Several projects have attempted to tackle the proliferation of good resources, but most often segmented into a particular type of resource. For example NIH’s iSearch COVID-19 portfolio (NIH OPA, 2020) aggregates scholarly articles as does the Kaggle COVID-19 Open Research Dataset Challenge (CORD-19) (Allen Institute For AI, 2020), but do not include clinical trials, datasets, or other types of resource. As a result, searching for resources across resource types becomes challenging.

In addition to challenges searching *between* resource types, the lack of standardization even *within* a particular type of resource remains a gap. The most organized efforts towards addressing this issue within a particular type of resource emerged from existing resource repositories which were able to pivot quickly, and curate COVID-19 content from their existing collections. Attempting to organize content from the disparate resources around the web and these pivoted collections highlighted the existing discrepancies in metadata standardization and magnified areas lacking standardization. For example, researchers involved in PubMed, which uses Medline citation standards, pivoted quickly to create LitCovid which follows the same standard (Chen, Allot and Lu, 2020). The preprint servers, bioRxiv and medRxiv follow their own minimal metadata standards and have their own list of COVID-19 related preprints (bioRxiv, 2021). The Medline and preprint standards have many overlapping metadata properties, but the overlapping properties may have different property names making it difficult to normalize and search through the metadata even though they describe the same type of resource. Similarly, National Clinical Trials Registry follows their own Protocol Registration and Results System (PRS) schema and has their own custom list of COVID-19 Clinical Trials which follows the same PRS standard (ClinicalTrials.gov, 2018), but these conventions are not followed by the WHO International Clinical Trials Registry Platform. Zenodo (Fava et al., 2020) and Figshare (Hyndman, 2020), which both enable export to multiple open data formats including json schema/schema.org, do not completely agree on the marginality, cardinality, and selection of the properties in profiles they use (European Organization For Nuclear Research, 2013)(Hahnel, 2018)(Schema.org, 2015).

In addition to challenges in finding and standardizing the metadata from COVID-19 resources, researchers also faced challenges in making openly available data more interpretable and shareable. Numerous sites and apps ingest and visualize epidemiological data from John Hopkins University (Center for Systems Science and Engineering, 2020) and The New York Times (The New York Times, 2020) to generate dashboards (Hejazi, 2020) (Global Change Data Lab and University of Oxford, 2020) (Mayo Clinic, 2020). However, these sources may vary in epidemiological factors provided, location information, and data granularity making it non-trivial to integrate altogether. Dashboards with interpretable visualizations can be difficult to explore, and those that can be explored can be difficult to link and share.

Leveraging our experience from building the NIAID Data Portal (Tsueng et al., 2022), we address the aforementioned challenges inherent in combining metadata from scattered and heterogeneous resources and making information more interpretable by building a website which consists of three parts: A searchable interface for a diverse, heterogeneous resources which we have collected and standardized (METADATA), an explorable tool to delve into epidemiological spatiotemporal trends. (DATA), and surveillance reports on SARS-CoV-2 variants and mutants (DATA). Consistent with best practices for FAIRness, our website includes programmatic access via APIs and a standardized interface built off schema.org. Daily updates ensure that site users have up-to-date information, essential in the midst of a constantly changing research landscape. We will discuss the standardization of metadata and the visualization of epidemiological data and provide a common use case for the site. Mutations, lineage, and variant reports are described elsewhere (Gangavarapu et al., 2022).

## Methods

### Schema development

The development of the schema for standardizing our collection of resources is as previously described (Cano et al., 2021). Briefly, we prioritized 5 classes of resources which had seen a rapid expansion at the start of the pandemic due to their importance to the research community: Publications, Datasets, Clinical Trials, Analysis, and Protocols. We identified the most closely related classes from schema.org and mapped their properties to available metadata from 2–5 of the most prolific sources. Additionally, we identified subclasses which were needed to support our main 5 classes and standardized the properties within each class. In addition to standardizing ready-to-harvest metadata, we created new properties which would support the linkage, exploration, and evaluation of our resources. Our schema was then refined as we iterated through the available metadata when assembling COVID-19 resources. The Outbreak schema is available at https://discovery.biothings.io/view/outbreak.

### Assembly of COVID-19 resources

The resource metadata pipeline for outbreak.info includes two ways to ingest metadata. First, metadata can be ingested from other resource repositories or collections using the BioThings SDK data plugins. For each resource repository/collection, a parser/data plugin enables automated import and updates from that resource. Second, metadata for individual resources can be submitted via an online form. To assemble the outbreak.info collection of resources, we collected a list of over a hundred separate resources on COVID-19 and SARS-CoV-2. This list (Supplemental Table X) included generalist open data repositories, biomedical-specific data projects including those recommended by the NIH (BioMedical Informatics Coordinating Committee, 2020) and NSF (National Science Foundation, 2013) to house open data, and individual websites we came across through search engines and other COVID-19 publications. Prioritizing those resources which had a large number of resources related to COVID-19, we selected an initial set of 2–3 sources per resource type to import into our collection. Given the lack of widespread repositories for Analysis Resources, only one source would be included in our initial import (Imperial College London). An Analysis resource is defined as a frequently-updated, web-based, data visualization/interpretation/analysis resource.

### Community curation of resource metadata

Resource plugins such as those used in the assembly of COVID-19 resources do not necessarily have to be built by our own team. We used the BioThings SDK (Lelong et al., 2021) and the Data Discovery Engine (Cano et al., 2021) so that individual resource collections can be added by writing BioThings plugins that conform to our schema. Expanding available classes of resources can be done easily by extending other schema.org classes via the DDE Schema Playground at https://discovery.biothings.io/schema-playground. Community contributions of resource plugins can be done via GitHub. In addition to contributing resource plugins for collections/repositories of metadata, users can enter metadata for individual resources via the automatic guides created by the Data Discovery Engine. To investigate potential areas of community contribution, we asked two volunteers to inspect 30 individual datasets sprinkled around the web and collect the metadata for these datasets. We compared the results between the two volunteers and their combined results were subsequently submitted into the collection via the Data Discovery Engine’s Outbreak Data Portal Guide at https://discovery.biothings.io/guide/outbreak/dataset. Improvements or updates for manually curated metadata can be submitted via GitHub pull requests.

### Community curation of searching, linkage, and evaluation metadata and scaling with machine learning

In an effort to enable improved searching and filtering, we developed a nested list of thematic or topic-based categories based on an initial list developed by LitCovid (Chen, Allot and Lu, 2020) with input from the infectious disease research community and volunteer curators. The list consists of 11 broad categories and 24 specific child categories. Whenever possible, sources with thematic categories were mapped to our list of categories in order to develop a training set for basic binary (in group/out group) classifications of required metadata fields such as (title, abstract and/or description). If an already-curated training set could not be found for a broad category, it would be created via an iterative process involving term/phrase searching on LitCovid, evaluating the specificity of the results, identifying new search terms by keyword frequency, and repeating the process. To generate training data for classifying resources into specific topic categories, the results from several approaches were combined. These approaches include keyword mapping from LitCovid, logical mapping from NCT Clinical Trials metadata, the aforementioned terms search iteration, and citizen science curation of Zenodo and Figshare datasets.

The efforts of our two volunteers suggested that non-experts were capable of thematically categorizing datasets, so we built a simple interface to allow citizen scientists to thematically classify the datasets that were available in our collection at that point in time. Each dataset was assigned up to 5 topics by at least three different citizen scientists. Citizen scientists were asked to prioritize specific topic categories over broader ones. The citizen science curation site can be found at https://curate.outbreak.info, the code for the site can be found at https://github.com/outbreak-info/outbreak.info-resources/tree/master/citsciclassify and the citizen science classifications at https://github.com/outbreak-info/topic_classifier/blob/main/data/subtopics/curated_training_df.pickle. These classifications have been incorporated into the appropriate datasets in our collection, and have been used to build our models for topic categorization. Basic in-group/out-group classification models were developed for each category using out-the-box functions and parameters available from SciKitLearn. The topic classifier can be found at https://github.com/outbreak-info/topic_classifier.

In addition to community curation of topic categorizations, we identified a citizen science effort, the COVID-19 Literature Surveillance Team (COVID19-LST), that was evaluating the quality of COVID-19 related literature. The COVID-19 LST consists of medical students, practitioners and researchers who evaluate publications on COVID-19 based on the Oxford Levels of Evidence criteria and write Bottom Line, Up Front summaries (Rah et al., 2020). With their permission, we integrated their outputs (daily reports/summaries, and evaluations) into our collection.

We further integrated our publications by linking preprints and their peer-reviewed versions. We performed separate Jaccard’s similarity calculations on the title/text and authors for preprint vs LitCovid Publications. We identified thresholds with high precision, low sensitivity and binned the matches into (expected match vs needs review). We also leveraged NLM’s pilot preprint program to identify and incorporate additional matches. The code used for the preprint-matching can be found at https://github.com/outbreak-info/outbreak_preprint_matcher. Expected matches were linked via the `correction` property in our schema.

### Harmonization and integration of resources, epidemiology, and genomics data

For epidemiology, we ingested data from John Hopkins University (JHU) and the New York Times. We normalized location information using geographic data from Natural Earth, World Bank, and the US Census Bureau. The code for the harmonization and integration of epidemiology data can be found at https://github.com/outbreak-info/biothings_covid19. The integration of genomics data from GISAID is discussed elsewhere (Gangavarapu et al., 2022). We built separate API endpoints for our resources (metadata resources API), epidemiology (epi data API) and genomics (genomics data API) using the BioThings SDK (Lelong et al., 2021). Data is available via our API at api.outbreak.info and through our R package, as described in Gangavarapu et al..

## Results

### Schema Development and COVID-19 resources available in outbreak.info

**Figure 2:**
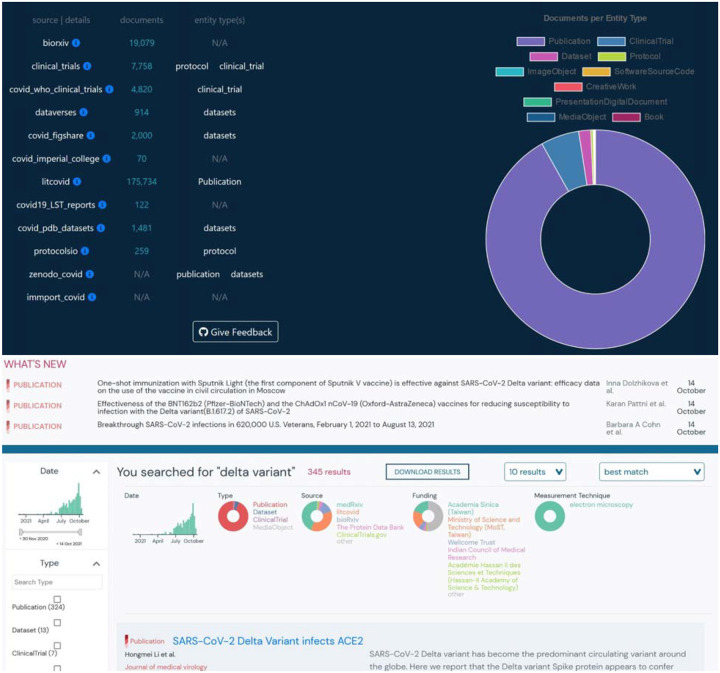
A. Distribution of resources by resource type and source. B. Heterogeneous and filterable resources (ie- publications, clinical trials, datasets, etc.) resulting from a single search of the phrase “Delta Variant”

Schema.org’s immense flexibility provides the necessary versatility to standardize metadata across the world wide web; however, excessive flexibility can make it challenging to standardize biomedical resources of different types. For example, publications typically use the ‘author’ property, while dataset providers typically prefer ‘creator’. Although both properties are valid for their respective schema.org classes, we normalized our schema to use ‘author’ for all 5 of our classes since we expected the volume of publications to dwarf all other classes of resources. Once our schema was developed, we created parsers (data plugins) to import compliant metadata from 14 resources. We assembled the data plugins into a single API via BioThings, and scheduled them to update on a daily basis to ensure up-to-date information. LitCovid, a source of Publications, provided the vast majority of entries into our resource library, followed by the preprint servers (bioRxiv and medRxiv)(Figure 2A).

Sources of certain metadata did not map readily to existing schema.org classes. For example, clinical trials registries like NCT have one general schema for both observational and interventional studies, while schema.org provides separate classes for each of these types of studies. Since it was a primary source of clinical trials metadata for our research library, we had to tailor the Outbreak Schema appropriately. Clinical Studies from NCT and WHO made up the next largest class of resources imported. Fortunately, many dataset repositories offered schema.org-compliant metadata, even if the repositories differed in the metadata fields that were available. Datasets were imported from Zenodo, Figshare, PDB, and Harvard Datasets; while Protocols were imported from Protocols.io and NCT Protocols. With a unified schema that could harmonize information across heterogeneous resource types, a single search (for example “delta variant”) can return relevant publications, datasets, clinical trials, and more. (Figure 2B)

### Acquisition of individual dataset metadata and collections of resource metadata via community curation

**Figure 3.**
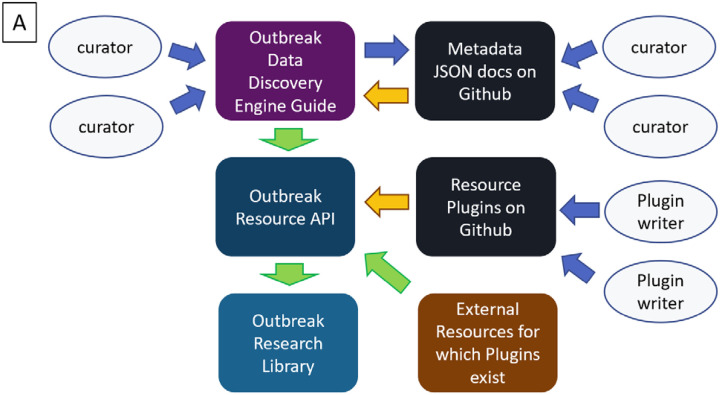

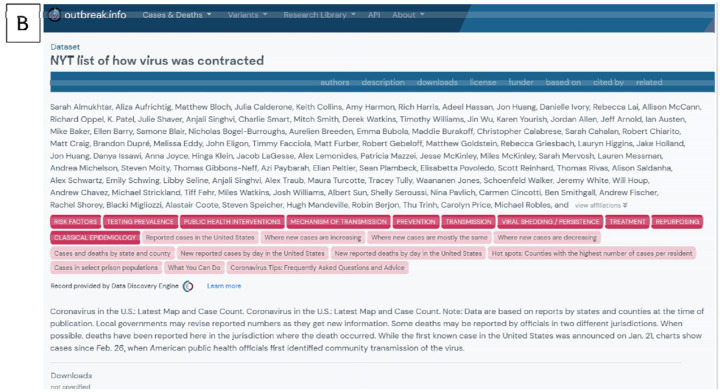
The community contributions. A- The community contribution pipeline for Outbreak’s Research Library. Curators may submit dataset metadata using the DDE built-in guide or from Github via the DDE. Python-savvy contributors can create parsers to contribute even more metadata via the BioThings SDK plugin architecture. A resource plugin allows the site to automatically ingest and update metadata from the corresponding external resource. Blue arrows indicate manual steps, yellow arrows indicate automatable steps after an initial set up, green arrows indicate completely automated steps. B- An example of detailed metadata curated by volunteers as it appears in the Research Library.

Given the diffuse and frequently changing nature inherent to biomedical resources, we built outbreak.info with the idea that it should be expanded with the participation of the community. Not only is finding and adding resources to the collection an onerous process, it also requires us to know the full landscape of resources on the internet. Furthermore, many resources cannot be readily parsed for metadata useful for linkage, exploration, and evaluation. We enabled contributions of resource metadata in a variety of ways (Figure 3A).

For single datasets, contributors can submit the metadata via Outbreak’s data submission guide on the Data Discovery Engine, which ensures that the curated metadata conforms to our schema. From there, it can be saved to Github, where it can be improved by other contributors via forking and pull requests. The Data Discovery Engine will automatically pass the information to the Outbreak Resources API where it is made discoverable with the Outbreak Research Library. Additionally, collections of standardized datasets, publications and other resources can be submitted to the Outbreak Resources API by contributing a resource plugin. Resource plugins are BioThings-compatible parsers which can be submitted by anyone with some python coding skills (Lelong et al., 2021). As seen in Figure 3B, community-contributed metadata can be exhaustively detailed. Although both of our volunteers provided values for many of the available metadata properties (name, description, topicCategories, keywords, etc.), one provided an extensive list of authors.

### Improved searching, linkage and evaluation of resources via community curation and machine learning

**Figure 4:**
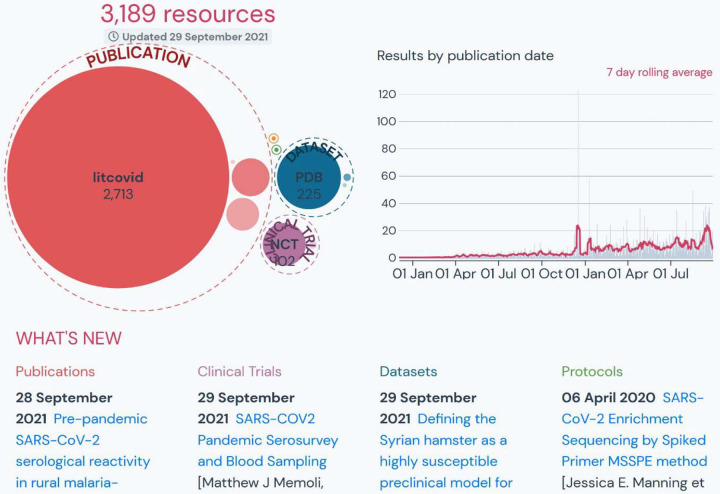
Visualizations of the data resulting from the example query in Supplemental Figure 1.

In addition to community-contributed metadata on individual resources, citizen scientists contributed classifications for categorizing the resources by topic and providing evaluations of resources. Pending implementation, users will be able to search the research library by topic category, filter for publications that have been evaluated by COVID-19 LST based on Oxford 2011 Levels of Evidence guidance, and sort results by altmetric score. Preprints can be linked to and de-duplicated from their peer-reviewed counterparts. The data can also be accessed via the resources API (Supplemental Figure 1), aggregated, analyzed, and visualized as seen in Figure 4. Figure 4 illustrates the number and breakdown of resources in the research library that have authors or funders affiliated with the National Institute of Allergy and Infectious Disease (NIAID).

### Harmonization and integration of resources, epidemiology, and genomics data

**Figure 5:**
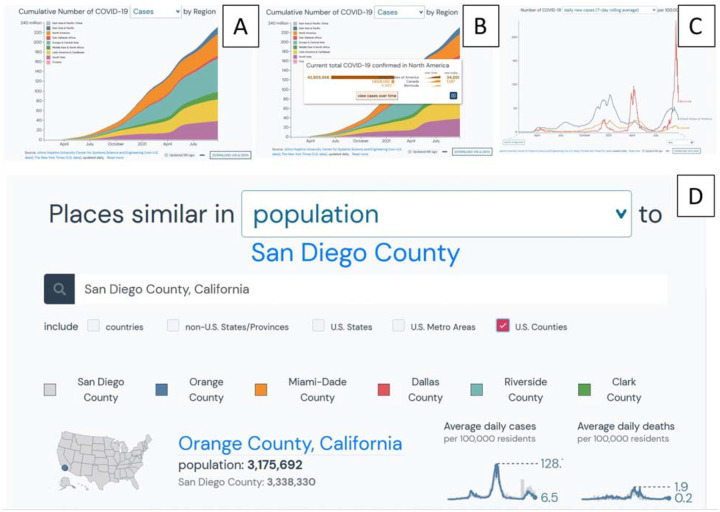

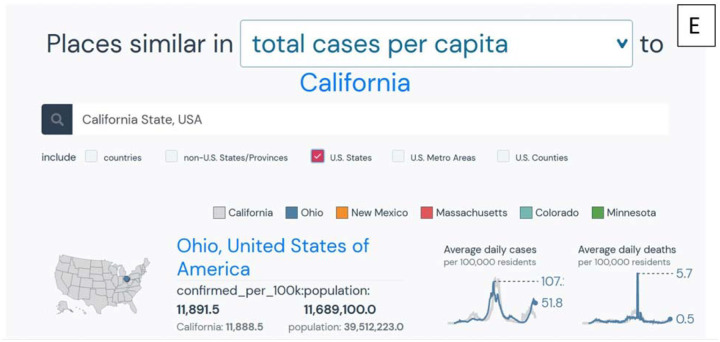
Overview of interactive visualizations for understanding, interpreting, and exploring epidemiological data. A- Cumulative cases by World Bank region, B- Regional details obtained by clicking on a region in A. C- Additional exploration of data in B. D-The comparison feature for finding regions similar in population or other epidemiological factors like total cases per capita (E).

In addition to collecting, standardizing, and importing metadata from multiple resources, outbreak.info integrates epidemiology data from Jon Hopkins University (JHU) and the New York Times with geographic data from Natural Earth and the US Census Bureau to create interactive, interpretable, and shareable visualizations. For example, users can explore the number of cases or deaths by World Bank regions (Figure 5A), click on a region to see the data by country within that region (Figure 5B), click to further compare other epidemiological measures between those countries (Figure 5C), or other locations of interest. Throughout the pandemic people have struggled to make meaningful comparisons that would help others understand the currently available data. Our interactive visualizations allow users to select a region and find comparable locations based on epidemiological factors such as population (Figure 5D), raw cases, cases per capita (Figure 5E), and more. Although there are numerous dashboards that have been built using the epidemiology data from JHU, our epidemiology interface in outbreak.info is built with the idea of supporting research. Its integration with the resource collection and the genomics data allows users to dig into the data, customize visualizations and export the results or access them via API. If a user wishes to share what they’ve learned, the interface allows for easy download of static snapshots of the customized visualization, as well as the corresponding data used to generate it. The integrated genomics data provided by GISAID can be used as described in the mutation reports paper, and are subject to the terms of use and methods described therein [ref].

### Accessing the data

**Figure 6:**
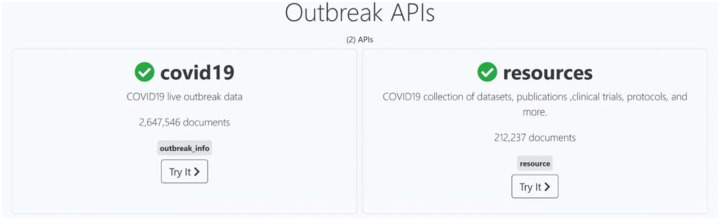
The epidemiology API endpoint and the resources API endpoint

In addition to providing a searchable interface for the data, interactive, interpretable and downloadable visualizations, the resources metadata, and epidemiology data can be accessed via their corresponding APIs (Figure 6) and via the R package. The APIs include a user-friendly interface (Supplemental Figure 1) for testing queries. Access to the genomics data is subject to GISAID’s terms of use. Use of the R package and access to the genomics data are described in the mutation reports paper (Gangavarapu et al., 2022).

## Discussion

Over the course of the COVID-19 outbreak, researchers have shared the outputs of their work at unprecedented levels –exacerbating existing issues with organizing outputs for meaningful search. These issues include the lack of structure and standardization (it’s hard to assemble), the lack of linkages (it’s hard to traverse meaningfully), frequent updates (it’s changing all the time), pipeline maintenance (original sources change as well) and data fragmentation (it’s everywhere). Further, the urgency of a pandemic requires that these issues be addressed quickly (need the information available ASAP), and in a scalable manner (need to be able to accommodate more and more data and its use). Launched within 2 months of the COVID-19 pandemic, outbreak.info is our attempt to address some of these issues.

To address the structure and standardization issue, we developed a schema and standardized metadata across different resources and ingested them into an accessible API and user-friendly search-and-filter, web-based interface. In addition to issues with standardizing and mapping metadata between resources, it is challenging to maintain a resource library that imports metadata from so many sources (pipeline maintenance issue), particularly when the metadata updates daily (update issue). Any changes to an external API or the way in which metadata is offered by an external site will necessitate a change in the parser. Fortunately, Outbreak’s resource API utilizes the BioThings SDK plugin architecture so errors in individual parsers may be addressed separately without affecting the availability of the API itself. Using the plugin architecture allows the introduction and maintenance of the individual resource parsers to be crowdsourced to any participant with some basic knowledge or python coding and a GitHub account. Although resource plugins allow outbreak.info to ingest large amounts of standardized metadata, there are still many individual datasets and research outputs scattered throughout the web (data fragmentation issue). Since it is not feasible for our team to locate, identify, and collect standardized metadata from these individual datasets and research outputs, we leveraged the Data Discovery Engine to enable crowdsourcing and citizen science participation in the curation of individual resource metadata.

Citizen scientists have played an active role in data collection (Birkin, Vasileiou, and Stagg 2021) (Rohwer Lab at San Diego State University, n.d.) and making information more accessible (Rah et al., 2020)(Allen Institute For AI, 2020) throughout the current pandemic. Given their ability to perform information extraction (Tsueng et al., 2020) and their immense contributions to classification tasks (Blickhan, Trouille, and Lintott, 2018), we incorporated citizen science contributions into the training data for classifying resources into our topic categories. Some resource aggregators have used clustering algorithms to categorize the entries in their resource libraries--though many only aggregate resources of a single type (ie-publications). We employed a different approach due to the heterogeneity of our resources, but our API is openly accessible, so anyone is welcome to apply clustering approaches to classify the entries. The research library and resources API has been used by others to monitor available research on the immune escape of variants, or create custom research digests. The Radx-Rad Data Coordination Center (https://www.radxrad.org) is utilizing the Outbreak API to collect articles for customized research digests for its partners. Using SearchOutbreak (https://searchoutbreak.netlify.app), users select topics based on information submitted from partners. These are turned into queries for the Outbreak api. These are stored in a database (airtable). Weekly the Outbreak API is queried, and new articles are added to the digests which are available at the website. A workflow sends an email to subscribed users. While these digests are not currently available to the public, they are expected to be available to the public in the future (Valentine D and Radx. 2021).

In addition to generating metadata values for improved searching and filtering, we encouraged/allowed linkages between resources in our schema. Improved linkages would enable more meaningful investigation of the data, but these linkages were largely unavailable from the original resource (linkage issue). For instance, ideally a publication about a clinical trial would link to its clinical trial record, protocols used to collect the data, datasets used in their analyses, and software code underlying the analyses. Challenges to include these linkages included: the lack of unique identifiers, inconsistent use of citation metadata fields between resources, and the lack of structured linkage metadata. Many Datasets and Analysis often don’t have unique identifiers, or aren’t referred to by their unique identifiers if they do have them. For example, the ONS Deaths Analysis does not have a unique identifier as assigned by Imperial College London, lacks any citation metadata fields, and instead mentions a potential linkage to an Imperial College London report in its mention of limitations (Imperial College COVID-19 Response Team, n.d.). Although preprints from bioRxiv and MedRxiv may have links to the corresponding peer-reviewed manuscript on the bioRxiv site, this information is not accessible via their API necessitating the use of algorithms to generate these links.

In the case of epidemiology data, much of the region-specific data harmonization and integration was already performed by our sources, though challenges in terminological harmonization, geospatial standardization and data scaling remained. For example, the definition of a “confirmed case” could vary between county and country, and changes in the definition during the course of the pandemic were challenging to address (Perhne, 2020)(John Hopkins University, 2020). Geographic boundaries could also be outdated or in need of normalization. For example, Basse-Normandie is an out-of-date name of a region in the genomics data that needed to be updated in order to map to the corresponding region in the epidemiology data.

Within the eighteen months since SARS-CoV-2 was first identified as the infectious agent of the COVID-19 pandemic, there have been over 170 million cases and nearly 4 million deaths. As those numbers continue to grow, so too does the research and understanding of the causes and consequences of the spread of this virus. Given that there will be other pandemics in the future, we demonstrate how open source software and tools can be used to rapidly build a standardized, searchable platform for exploring COVID-19 epidemiological data, research outputs, and genomics data. Our platform, outbreak.info, seeks to make COVID-19 data more findable, accessible, interoperable, reusable and interpretable by addressing many data management issues exposed by an urgent and frequently-changing situation. It is used by a wide variety of professionals including journalists, members of the medical and public health communities, students, and biomedical researchers (blog.outbreak.info). On average, the site receives over a thousand hits per day and its visualizations are shared frequently across social media platforms like Twitter.

## Conclusions

We have built a standardized, searchable platform to discover and explore COVID-19 research outputs and data. We address many of the challenges faced when assembling a collection of heterogeneous research outputs and data into a searchable platform. We demonstrate how such a platform could be launched within 2 months of a pandemic using existing scalable, open source software development kits and tools.

## Supplementary Material

1
