# Outbreak.info Research Library: A standardized, searchable platform to discover and explore COVID-19 resources

**DOI:** 10.1101/2022.01.20.477133

**Published:** 2022-06-02

**Authors:** Ginger Tsueng, Julia L. Mullen, Manar Alkuzweny, Marco Cano, Benjamin Rush, Emily Haag, Outbreak Curators, Alaa Abdel Latif, Xinghua Zhou, Zhongchao Qian, Emory Hufbauer, Mark Zeller, Kristian G. Andersen, Chunlei Wu, Andrew I. Su, Karthik Gangavarapu, Laura D. Hughes

**Affiliations:** 1 Department of Integrative, Structural and Computational Biology, The Scripps Research Institute, La Jolla, CA 92037, USA; 2 Department of Biological Sciences, University of Notre Dame, Notre Dame, IN 46556, USA; 3 Ocuvera, Lincoln, NE 68512, USA; 4 Department of Immunology and Microbiology, The Scripps Research Institute, La Jolla, CA 92037, USA; 5 Scripps Research Translational Institute, La Jolla, CA 92037, USA; 6 Department of Molecular Medicine, The Scripps Research Institute, La Jolla, CA 92037, USA; 7 Department of Human Genetics, David Geffen School of Medicine, University of California Los Angeles, Los Angeles, CA 90095, USA

## Abstract

To combat the ongoing COVID-19 pandemic, scientists have been conducting research at breakneck speeds, producing over 52,000 peer-reviewed articles within the first year. To address the challenge in tracking the vast amount of new research located in separate repositories, we developed outbreak.info Research Library, a standardized, searchable interface of COVID-19 and SARS-CoV-2 resources. Unifying metadata from twelve repositories, we assembled a collection of over 270,000 publications, clinical trials, datasets, protocols, and other resources as of May 2022. We used a rigorous schema to enforce consistency across different sources and resource types and linked related resources. Researchers can quickly search the latest research across data repositories, regardless of resource type or repository location, via a search interface, public API, and R package. Finally, we discuss the challenges inherent in combining metadata from scattered and heterogeneous resources and provide recommendations to streamline this process to aid scientific research.

## Introduction

**Figure 1.**
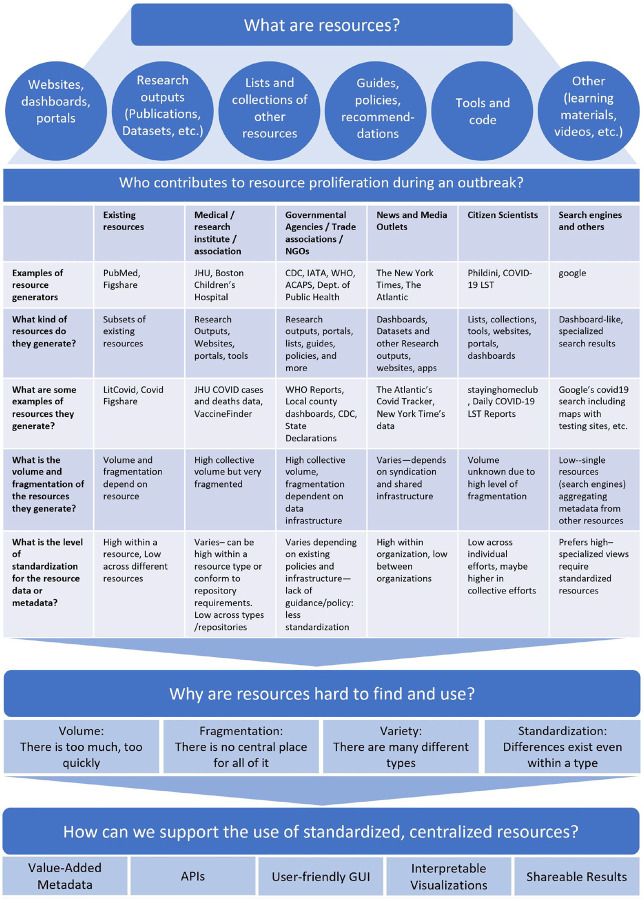
What are resources, who contributes to the proliferation of resources, why are resources difficult to find and use, and how can we support their use?

In early January 2020, SARS-CoV-2 was identified as the virus responsible for a series of pneumonia cases with unknown origin in Wuhan, China^1^. As the virus quickly spread all over the world, the global scientific community began to study the new virus and disease, resulting in the rapid release of research outputs (such as publications, clinical trials, datasets) and resources (i.e. research outputs, websites, portals and more). The frequently uncoordinated generation and curation of resources by different types of resource generators (such as government agencies, NGOs, research institutes, etc.) exacerbate four factors that make finding and using resources a challenge: volume, fragmentation, variety, and standardization (Figure 1). These four factors hamper the ability for researchers to discover these resources, and consequently, impede the translation of these protocols, data, and insights into a synthesized understanding of the virus to help combat the pandemic.

For example, the volume of peer-reviewed articles from a single resource (LitCovid) has grown from about 52,000 published within the first twelve months to over 250,000 as of June 2022. Since April 2020, over 1,000 different research outputs have been published on a weekly basis, spanning new protocols, datasets, clinical trials, as well as publications. The rapid proliferation of resources could be manageable if there were a centralized repository for finding them, but none exists. In addition to research outputs like scientific literature, researchers, public health officials, media outlets, and concerned communities independently developed websites providing highly localized or specialized information on infection rates^2,3^, prevention policies^4,5,6^, and travel restrictions^7^ resulting in a fragmented landscape of very different types of resources (Figure 1).

The volume and fragmentation issues were immediately obvious. Lacking alternate solutions for addressing these issues, individual and community efforts for curating these resources were created via shared Google spreadsheets^8,9,10^ to aid in discoverability. However, the sheets were not a scalable solution and usually lacked sufficient metadata for describing resources, with the exception of Navarro and Capdarest-Arest. Several projects have attempted to address the volume and fragmentation issues, but were most often focused on a single type of resource. For example, NIH’s iSearch COVID-19 portfolio^11^ and the Kaggle COVID-19 Open Research Dataset Challenge (CORD-19)^12^ aggregate scholarly articles, but do not include clinical trials, datasets, or other types of resources.

Compounding search issues caused by the variety of resource types, there has been a longstanding lack of standardization even *within* a particular type of resource. Existing resource repositories which were able to pivot quickly and curate COVID-19 content from their collections utilized pre-existing metadata standards. For example, researchers involved in PubMed, which uses Medline citation standards, shifted quickly to create LitCovid^13^ which follows the same standard. Similarly, the National Clinical Trials Registry has their own custom list of COVID-19 Clinical Trials which follows their own Protocol Registration and Results System (PRS) schema^14^, but these conventions are not followed by the WHO International Clinical Trials Registry Platform. Zenodo^15^ and Figshare^16^, which both enable export to multiple open data formats including schema.org, do not completely agree on the marginality, cardinality, and selection of the properties in profiles they use^17,18,19^.

Once the issues of volume, fragmentation, variety, and standardization of resources are addressed, accessibility of the resulting resources for reuse must be addressed. Standardized, centralized resources are of no value if researchers are not able to leverage them. Researchers seeking to process information *en masse* will need an API, while researchers seeking to browse and explore will prefer a user-friendly interface. APIs themselves are less useful without a means of understanding the underlying metadata/data (such as documentation or a GUI), and a user-friendly search portal will be less useful without the inclusion of value-added metadata (such as ones supporting search/filter, linkage and exploration, or qualitative evaluation) for improving resource discovery and interpretation. Interpretability of metadata/data is influenced by the order in which information is presented. To address this challenge, the user interface must encourage exploration which gives users control over the information flow to suit their needs. Lastly, if a user has been able to successfully leverage the standardized, centralized resources, they should be able to easily save and share the results of their efforts.

We address the aforementioned challenges inherent in combining metadata from disparate and heterogeneous resources and making information more interpretable by building outbreak.info, a website which integrates a searchable interface for a diverse, heterogeneous resources which we have collected and standardized (metadata) with surveillance reports on SARS-CoV-2 variants and mutants (data). Following implementation considerations for FAIRness^20^, our website includes programmatic access via APIs and a standardized metadata interface built off schema.org. Daily updates ensure that site users have up-to-date information, essential in the midst of a constantly changing research landscape. Based on our experience unifying metadata across repositories, we will discuss issues with centralizing, standardizing, and returning resource metadata, epidemiological data, and supporting the use of the metadata/data. In a companion piece, we present our efforts to develop genomic reports to scalably and dynamically track SARS-CoV-2 variants^21^.

## Results

### Standardizing metadata through a schema harmonizing a variety of resource types.

**Figure 2.**
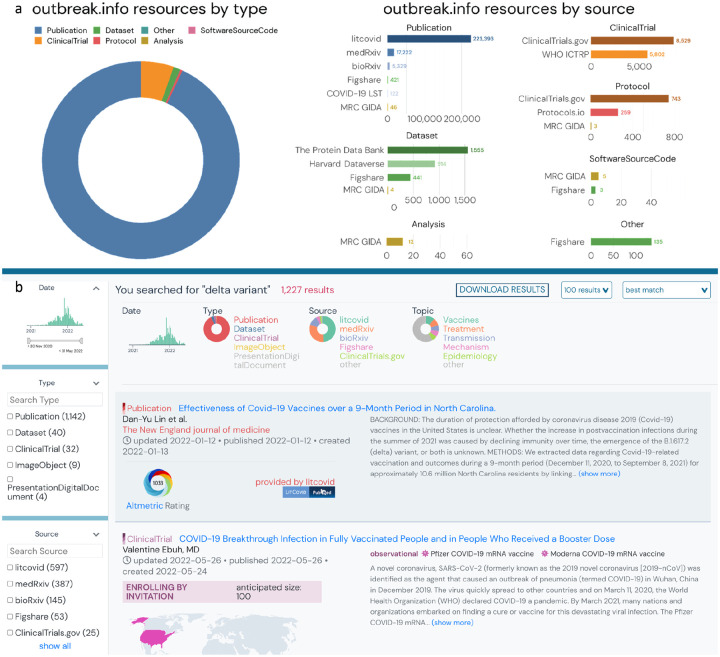
Supporting resource centralization and standardization by developing a harmonizing schema. **a**, Distribution of resources by resource type and source. Note that the x-axis in the bar graphs have different scales. **b**, Heterogeneous and filterable resources (i.e. publications, clinical trials, datasets, etc.) resulting from a single search of the phrase “Delta Variant”.

We address issues with metadata variety, standardization, and fragmentation by developing a harmonized schema. Schema.org provides a framework to standardize metadata for many different types of data found on the world wide web. However, these standards are not preserved across different types of data. For example, publication providers like PubMed typically use the ‘author’ property in their metadata, while dataset providers like Figshare and Zenodo are compliant with the DataCite schema and typically prefer ‘creator’. Although both properties are valid for their respective schema.org classes, we normalized our schema to use ‘author’ for all 5 of our classes since we expected the volume of publications to dwarf all other classes of resources. We developed a schema that encompassed five types of resources based on their proliferation at the beginning of the pandemic and their importance to the research community: Publications, Datasets, Clinical Trials, Analysis, and Protocols. We added this schema to the Schema Registry of the Data Discovery Engine (DDE)^22^, a project to share and reuse schemas and register datasets according to a particular schema. Using this schema we ingested and harmonized metadata from an initial set of twelve key resources: LitCovid (Publications), bioRxiv/medRxiv (Publications), ClinicalTrials.gov (NCT) (ClinicalTrials), WHO International Clinical Trials Registry Platform (WHO ICTRP) (ClinicalTrials), Figshare (Datasets, Publications, and more), Zenodo (Datasets, Publications, and more), MRC Centre for Global Infectious Disease Analysis (Analyses, Publications, and More), Protocols.io (Protocols), Protein Data Bank (PDB) (Datasets), Harvard Dataverse (Datasets), COVID-19 Literature Surveillance Team (Publications), and ImmPort (Datasets) (Figure 2a).

Sources of certain metadata did not map readily to existing schema.org classes. For example, clinical trials registries like NCT have one general schema for both observational and interventional studies, while schema.org provides separate classes for each of these types of studies. Since NCT was a primary source of clinical trials metadata for our research library, we tailored the Outbreak schema based on the combined general NCT schema. Fortunately, many dataset repositories offered schema.org-compliant metadata, even if the repositories differed in the metadata fields that were available. Dataset metadata was harvested from Zenodo, Figshare, PDB, and Harvard Datasets, while Protocols were imported from Protocols.io and NCT Protocols. Once our schema was developed, we created parsers (data plugins) to import and standardize metadata from our initial set of resources. We assembled the data plugins into a single API via BioThings SDK^23^, and scheduled them to update on a daily basis to ensure up-to-date information. By leveraging the BioThings SDK, we developed a technology stack that addresses the fragmentation issue by easily integrating metadata from different pre-existing resources. With a unified schema that harmonizes information across heterogeneous resource types, a single search (for example “delta variant”) to our API can return relevant publications, datasets, clinical trials, and more (Figure 2b).

### Enabling community curation and metadata submission to address fragmentation and standardization issues.

**Figure 3.**
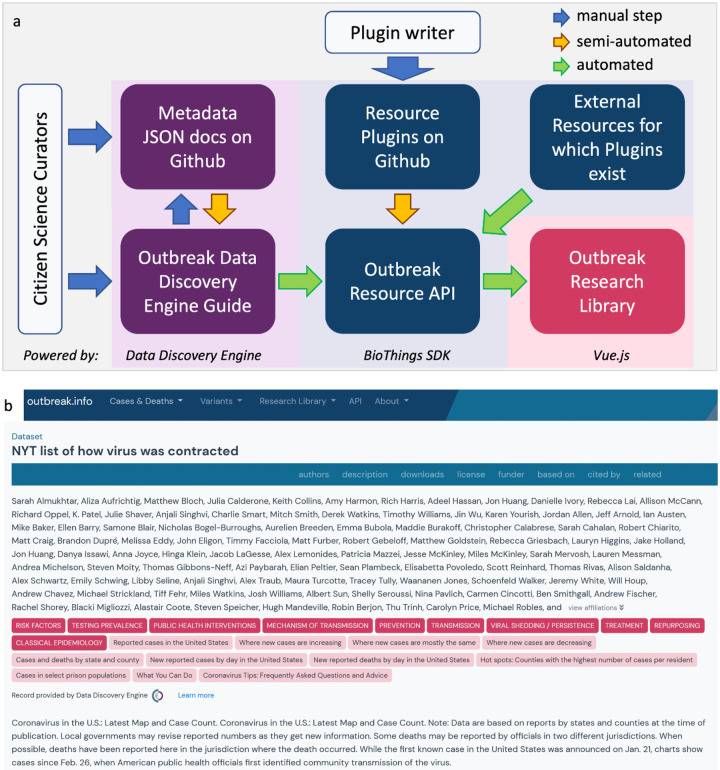
Aggregating resource metadata by leveraging community contributions. **a**, The community contribution pipeline and technology stack for outbreak.info’s Research Library. Curators may submit dataset metadata using the DDE built-in guide or from GitHub via the DDE/BioThings SDK. Pythonsavvy contributors can create parsers to contribute even more metadata via the BioThings SDK plugin architecture. A resource plugin allows the site to automatically ingest and update metadata from the corresponding external resource. Blue arrows indicate manual steps, yellow arrows indicate automatable steps after an initial set up, green arrows indicate completely automated steps. **b**, An example of a detailed metadata record manually-curated by volunteers as it appears in the Research Library.

At the start of the pandemic many curation efforts were neither coordinated, standardized, or easy to find; however, these efforts served an important role in organizing information early on. Given the highly-fragmented, diffuse and frequently changing nature inherent to biomedical resources, we built outbreak.info with the idea that it should be expanded with the participation of the community. Not only is finding and adding resources to the collection an onerous process, it also requires us to know the full landscape of resources on the internet. Furthermore, many resources do not collect metadata useful for linkage, exploration, and evaluation in machine-readable formats. We enabled community-based contributions of resource metadata in a variety of ways (Figure 3a).

For single datasets, contributors can submit the metadata via outbreak.info’s dataset submission guide on the Data Discovery Engine, which ensures that the curated metadata conforms to our schema. From there, it can be saved to GitHub, where it can be improved by other contributors via forking and pull requests. The DDE automatically passes the information to the outbreak.info Resources API where it is made discoverable with the Research Library. We demonstrated its utility by asking two volunteers to annotate metadata from thirty different individual resources from across the internet and submitted the metadata for integration via the DDE. As seen in Figure 3b, community-contributed metadata using the DDE is standardized and can be exhaustively detailed. Although both of our volunteers provided values for many of the available metadata properties (name, description, topicCategories, keywords, etc.), one provided an extensive list of authors. Using the BioThings SDK in conjunction with the DDE allows us to centralize and leverage individualized curation efforts that often occur at the start of a pandemic. Additionally, collections of standardized datasets, publications and other resources can be submitted to the Outbreak Resources API by contributing a resource plugin. Resource plugins are BioThings-compatible Python scripts to harvest metadata from a source and standardize it to our schema; these parsers can be submitted by anyone with Python coding skills^23^. Our community contribution pipeline allows us to quickly and flexibly integrate the uncoordinated data curation efforts, particularly apparent at the start of the pandemic (Supplemental Figure 1).

### Improving searching, linkage and evaluation of resources to support exploration

Centralizing and standardizing the resources does not automatically make the resources explorable to a user. While centralizing and standardizing allows for search, aggregation and some filtering; additional metadata and a user-friendly interface is needed to allow thematic browsing/filtering and to enable iterative traversal from query to search result to refined query and vice versa. To support resource exploration and interpretation, we added properties (value-added metadata) to every class in our schema that would support searching/filtering/browsing (topicCategories), linkage/exploration (correction, citedBy, isBasedOn, isRelatedTo), and interpretation (qualitative evaluations) of resources.

We selected these properties based on pre-existing citizen science and resource curation activities, suggesting their value in promoting discoverability. For example, citizen scientists categorized resources in their lists/collections by type (Dataset, Clinical Trials, etc.) in their outputs^10^ or area of research (Epidemiological, Prevention, etc.)^24^ as they found these classifications helpful for searching, filtering, and browsing their lists/collections. They also evaluated the level of evidence provided by these resources in order to improve its interpretability (i.e. understanding the credibility/quality of the resource)^24^. Existing repositories such as LitCovid also organized information to enhance browsability, but these efforts were often not captured in the metadata. For instance, LitCovid organized publications into eight research areas such as Treatments or Prevention, but these classifications are not available in the actual metadata records for each publication. To obtain these classifications from LitCovid, subsetted exports of identifiers must be downloaded from LitCovid and then mapped to the metadata records from PubMed.

**Figure 4.**
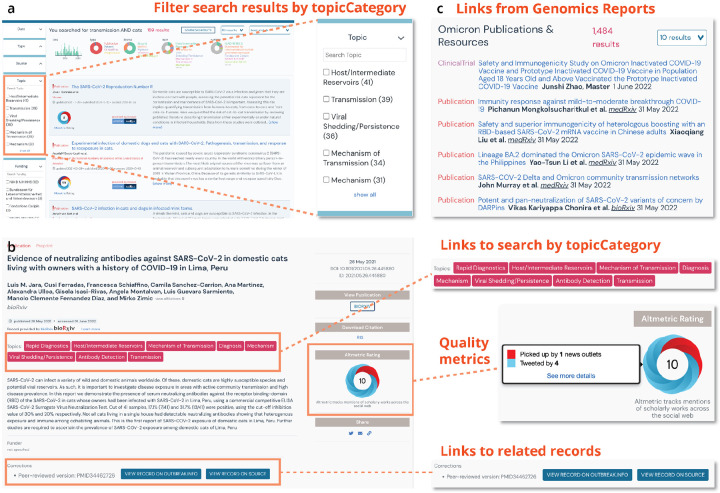
Enabling exploration of the resources. **a**, Selectable options for filtering results by topic category or other facets enhance searchability and exploration from the search results view. **b**, Links to other records or to additional potential searches of interest enabling further exploration from a record view. **c**, Links from the Omicron Variant report to related resources.

To classify resources by topicCategory and improve search/browse/filtering capabilities in our user interface, we used a combination of existing work (LitCovid) and human curation to augment that categorization to provide higher specificity of topics and to extend to new types of data (datasets, clinical trials). We applied out-the-box logistic regression, multinomial naive bayes, and random forest algorithms to create models for classifying each resource as belonging or not belonging to each topic. These three algorithms were found to perform best on this binary classification task using out-the-box tests. For example, if a user wants to browse for all resources (or filter down search results) related to the prevention of COVID-19, they can select the appropriate topicCategory in the search/search results view of the resources (Figure 4a). Users can also easily traverse from a view of a resource record to start a new search by clicking on a topicCategory of interest (Figure 4b). We further enable exploration by populating the linkage properties (corrections, citedBy, isBasedOn, isRelatedTo) from citation metadata (whenever possible), corrections metadata (from LitCovid, when available), and via an algorithm for matching peer-reviewed papers in LitCovid with their corresponding preprints in bioRxiv/medRxiv. Together with the corrections metadata from LitCovid, the algorithm has matched over 2,600 peer-reviewed articles with their corresponding preprints, enabling users to follow from a publication record from LitCovid to a publication record in bioRxiv/MedRxiv (Figure 4b).

Once a user has found a record of interest, they might wonder about the credibility of the resource. To populate resource evaluations so that users can assess the quality of a resource and tailor their interpretation accordingly, we leveraged the Oxford 2011 Levels of Evidence annotations generated by the COVID-19 Literature Surveillance (COVID-19 LST) team^24^ as well as Digital Science’s Altmetrics^25^. These evaluations are currently visible in the search results, and in the future, we will enable users to further filter or sort search results by some measurement of quality (i.e. Altmetrics: degree of access, or COVID-19 LST: level of evidence). Lastly, we integrated resources with data and analyses we curated to track SARS-CoV-2 variants^21^. Researchers can seamlessly traverse from a specific variant report like Omicron to resources on that variant to help understand its behavior (Figure 4c). In the absence of a centralized search interface with linked records, a similar attempt to explore resources outside the outbreak.info portal would require extensive manual searching from multiple different sites (Supplemental Figure 2), each with their own interfaces and corresponding search capabilities.

## Discussion

Over the course of the COVID-19 outbreak, researchers have shared the results of their work at unprecedented levels – exacerbating existing issues in resource volume, fragmentation, variety, and standardization. These issues make it challenging to assemble, traverse, and maintain up-to-date resources. Further, the urgency of a pandemic requires that these issues be addressed quickly, and in a scalable manner to be able to accommodate more data flexibly. We launched outbreak.info within 2 months of the start of the COVID-19 pandemic to address these issues and to highlight barriers in rapidly sharing research outputs in the midst of a pandemic.

To address the structure and standardization issue, we developed a standardized schema, integrated metadata from different resources into an accessible API, and created a user-friendly search-and-filter, web-based interface. In addition to difficulties standardizing inconsistent metadata models between resources, it is also challenging to maintain a resource library that imports metadata from so many sources, particularly when the metadata updates daily and is prone to change structure. Any changes to the upstream metadata offered by an external site necessitates a change in the parser which imports them. The resource API utilizes the BioThings SDK plugin architecture to handle errors in individual parsers without affecting the availability of the API itself. Using the plugin architecture also allows the creation and maintenance of the individual resource parsers to be crowdsourced to anyone with basic Python knowledge and a GitHub account. Although resource plugins allow outbreak.info to ingest large amounts of standardized metadata, there are still many individual datasets and research outputs scattered throughout the web which are not located in large repositories. Since it is not feasible for one team to locate, identify, and collect standardized metadata from these individual datasets and research outputs, we leveraged the Data Discovery Engine to enable crowdsourcing and citizen science participation in the curation of individual resource metadata.

At the onset of our data harvesting and harmonization efforts, we focused on creating a unified search interface backed by a common schema.org-based schema. With an extendable pipeline in place, we focused next on augmenting the existing metadata by adding properties to help researchers find information more quickly: topic categorization to group related research, resource linking to connect related entities along the data lifecycle from data generation through publication, and integrating external evaluations of the research trustworthiness using a combination of human curation and automated methods.

Citizen scientists have played an active role in data collection^26,27^ and making information more accessible^12,24^ throughout the current pandemic. Given their ability to perform information extraction^28^ and their immense contributions to classification tasks^29^, we incorporated citizen science contributions into the training data for classifying resources into our topic categories. Some resource aggregators have used clustering algorithms to categorize the entries in their resource libraries, though many only aggregate resources of a single type (i.e. publications). We employed a different approach due to the heterogeneity of our resources, but our API is openly accessible, so anyone is welcome to apply clustering approaches to classify the entries.

In addition to generating metadata values for improved searching and filtering, we enabled linkages between resources in our schema. For instance, ideally a publication about a clinical trial would link to its clinical trial record, protocols used to collect the data, datasets used in their analyses, and software code underlying the analyses to enable a more meaningful understanding of this trial. However, these connections rarely exist within the metadata; as a result, we have generated linkages between preprints and peer-reviewed publications, and plan to create more linkages between other resource types. Challenges to include these linkages included: the lack of unique identifiers, inconsistent use of citation metadata fields between resources, and the lack of structured linkage metadata. For example, the ONS Deaths Analysis does not have a unique identifier as assigned by Imperial College London, lacks any citation metadata fields, and instead mentions a potential linkage to an Imperial College London report in its mention of limitations^30^. Although preprints from bioRxiv^31^ and medRxiv may have links to the corresponding peer-reviewed manuscript on the bioRxiv site, this information is not accessible via their API, necessitating the use of algorithms to generate these links.

As a result of this centralization, standardization, and linkage, the outbreak.info Research Library and resources API has been widely used by the external community, including journalists, members of the medical and public health communities, students, and biomedical researchers^32^. For instance, the Radx-Rad Data Coordination Center (https://www.radxrad.org) is utilizing the Outbreak API to collect articles for customized research digests for its partners. Using the Radx-Rad SearchOutbreak app (https://searchoutbreak.netlify.app), users select topics based on information submitted from partners. These are turned into queries for the Outbreak API, and every week, new articles are added to the digests which are available at the website. A workflow sends an email to subscribed users. These digests are not currently available to the public but are expected to be released publicly in the future^33^. Overall, the site receives over a thousand hits per day on average and its visualizations are shared frequently across social media platforms like Twitter.

While we have developed a framework for addressing resource volume, fragmentation, and variety that can be applicable to future pandemics, our efforts during this framework exposed additional limitations in how data and metadata are currently collected and shared. Researchers have embraced pre-publications, but resources (especially datasets and computational tools) needed to replicate and extend research results are not linked in ways that are discoverable. Although many journals and funders have embraced dataset and source code submission requirements, the result is that the publication of datasets and software code are still heavily based in publications instead of in community repositories with well-described metadata to promote discoverability and reuse. In the outbreak.info Research Library, the largest research output by far is publications, while dataset submission lags in standardized repositories encouraged by the NIH such as ImmPort, Figshare, and Zenodo. We hypothesize that this disparity between pre-print and data sharing reflects the existing incentive structure, where researchers are rewarded for writing papers and less for providing good, reusable datasets. Ongoing efforts to improve metadata standardization and encourage schema adoption (such as the efforts in the Bioschemas community) will help make resources more discoverable in the future – provided researchers adopt and use them. For this uptake to happen, fundamental changes in the incentive structure for sharing research outputs may be necessary.

Within the eighteen months since SARS-CoV-2 was first identified as the infectious agent of the COVID-19 pandemic, there have been over 170 million cases and nearly 4 million deaths. As those numbers continue to grow, so too does the research and understanding of the causes and consequences of the spread of this virus. Given that there will be other pandemics in the future, we demonstrate how we built and launched an extendable and searchable platform for exploring COVID-19 research outputs and genomics data within two months of the pandemic. We address many of the challenges faced when assembling a collection of heterogeneous research outputs and data into a searchable platform. Our platform, outbreak.info, seeks to make COVID-19 data more findable, accessible, interoperable, reusable and interpretable by addressing many data management issues exposed by an urgent and frequently-changing situation. Our site is used by a wide variety of professionals including journalists, members of the medical and public health communities, students, and biomedical researchers^32^. On average, the site receives over a thousand hits per day and its visualizations are shared frequently across social media platforms like Twitter. This platform is also easily extensible to add new metadata sources, allowing the Research Library to grow with the pandemic as research changes.

## Methods

### Schema development

The development of the schema for standardizing our collection of resources is as previously described^22^. Briefly, we prioritized five classes of resources which had seen a rapid expansion at the start of the pandemic due to their importance to the research community: Publications, Datasets, Clinical Trials, Analysis, and Protocols. We identified the most closely related classes from schema.org and mapped their properties to available metadata from 25 of the most prolific sources. Additionally, we identified subclasses which were needed to support our main five classes and standardized the properties within each class. In addition to standardizing ready-to-harvest metadata, we created new properties which would support the linkage, exploration, and evaluation of our resources. Our schema was then refined as we iterated through the available metadata when assembling COVID-19 resources. The Outbreak schema is available at https://discovery.biothings.io/view/outbreak.

### Assembly of COVID-19 resources

The resource metadata pipeline for outbreak.info includes two ways to ingest metadata. First, metadata can be ingested from other resource repositories or collections using the BioThings SDK data plugins. For each resource repository/collection, a parser/data plugin enables automated import and updates from that resource. Second, metadata for individual resources can be submitted via an online form. To assemble the outbreak.info collection of resources, we collected a list of over a hundred separate resources on COVID-19 and SARS-CoV-2. This list (Supplemental Table 1) included generalist open data repositories, biomedical-specific data projects including those recommended by the NIH^34^ and NSF^36^ to house open data, and individual websites we came across through search engines and other COVID-19 publications. Prioritizing those resources which had a large number of resources related to COVID-19, we selected an initial set of 2–3 sources per resource type to import into our collection. Given the lack of widespread repositories for Analysis Resources, only one source would be included in our initial import (Imperial College London). An Analysis resource is defined as a frequently-updated, web-based, data visualization, interpretation, and/or analysis resource.

### Community curation of resource metadata

Resource plugins such as those used in the assembly of COVID-19 resources do not necessarily have to be built by our own team. We used the BioThings SDK^23^ and the Data Discovery Engine^22^ so that individual resource collections can be added by writing BioThings plugins that conform to our schema. Expanding available classes of resources can be done easily by extending other schema.org classes via the DDE Schema Playground at https://discovery.biothings.io/schema-playground. Community contributions of resource plugins can be done via GitHub. In addition to contributing resource plugins for collections/repositories of metadata, users can enter metadata for individual resources via the automatic guides created by the Data Discovery Engine. To investigate potential areas of community contribution, we asked two volunteers to inspect 30 individual datasets sprinkled around the web and collect the metadata for these datasets. We compared the results between the two volunteers and their combined results were subsequently submitted into the collection via the Data Discovery Engine’s Outbreak Data Portal Guide at https://discovery.biothings.io/guide/outbreak/dataset. Improvements or updates for manually curated metadata can be submitted via GitHub pull requests.

### Community curation of searching, linkage, and evaluation metadata and scaling with machine learning

In an effort to enable improved searching and filtering, we developed a nested list of thematic or topic-based categories based on an initial list developed by LitCovid^13^ with input from the infectious disease research community and volunteer curators. The list consists of 11 broad categories and 24 specific child categories. Whenever possible, sources with thematic categories were mapped to our list of categories in order to develop a training set for basic binary (in group/out group) classifications of required metadata fields such as (title, abstract and/or description). If an already-curated training set could not be found for a broad category, it would be created via an iterative process involving term/phrase searching on LitCovid, evaluating the specificity of the results, identifying new search terms by keyword frequency, and repeating the process. To generate training data for classifying resources into specific topic categories, the results from several approaches were combined. These approaches include direct mapping from LitCovid research areas, keyword mapping from LitCovid, logical mapping from NCT Clinical Trials metadata, the aforementioned terms search iteration, and citizen science curation of Zenodo and Figshare datasets.

The efforts of our two volunteers suggested that non-experts were capable of thematically categorizing datasets, so we built a simple interface to allow citizen scientists to thematically classify the datasets that were available in our collection at that point in time. Each dataset was assigned up to 5 topics by at least three different citizen scientists. Citizen scientists were asked to prioritize specific topic categories over broader ones. 90 citizen scientists participated in classifying 500 datasets pulled from Figshare and Zenodo. The citizen science curation site was originally hosted at https://curate.outbreak.info, the code for the site can be found at https://github.com/outbreak-info/outbreak.info-resources/tree/master/citsciclassify and the citizen science classifications at https://github.com/outbreakinfo/topic_classifier/blob/main/data/subtopics/curated_training_df.pickle. These classifications have been incorporated into the appropriate datasets in our collection, and have been used to build our models for topic categorization. Basic in-group/out-group classification models were developed for each category using out-the-box logistic regression, multinomial naive bayes, and random forest algorithms available from SciKitLearn. The topic classifier can be found at https://github.com/outbreak-info/topic_classifier.

In addition to community curation of topic categorizations, we identified a citizen science effort, the COVID-19 Literature Surveillance Team (COVID19-LST), that was evaluating the quality of COVID-19 related literature. The COVID-19 LST consists of medical students, practitioners and researchers who evaluate publications on COVID-19 based on the Oxford Levels of Evidence criteria and write Bottom Line, Up Front summaries^24^. With their permission, we integrated their outputs (daily reports/summaries, and evaluations) into our collection.

We further integrated our publications by adding structured linkage metadata, connecting preprints and their peer-reviewed versions. We performed separate Jaccard’s similarity calculations on the title/text and authors for preprint vs LitCovid Publications. We identified thresholds with high precision, low sensitivity and binned the matches into (expected match vs needs review). We also leveraged NLM’s pilot preprint program to identify and incorporate additional matches. The code used for the preprint-matching can be found at https://github.com/outbreak-info/outbreak_preprint_matcher. Expected matches were linked via the `correction` property in our schema.

### Harmonization and integration of resources and genomics data

The integration of genomics data from GISAID is discussed elsewhere^21^. We built separate API endpoints for our resources (metadata resources API) and genomics (genomics data API) using the BioThings SDK^23^. Data is available via our API at api.outbreak.info and through our R package, as described in Gangavarapu et al.

## Supplementary Material

Supplement 1

Supplement 2
