# Supplementary material for: Outbreak.info Research Library: A standardized, searchable platform to discover and explore COVID-19 resources
Source: bioRxiv. 2022 May 3:2022.01.20.477133. Preprint. [Version 3] doi: 10.1101/2022.01.20.477133 (PMC8820656; doi:10.1101/2022.01.20.477133)
Supplement: Supplementary file 1 [file media-1.docx]

Supplemental Figure 1: Example resource-generation pipelines observed within the first month of the pandemic, resulting in a highly-fragmented, non-standardized resource landscape that is difficult to search, centralize and leverage.

Supplemental Figure 2- Example resource exploration pathway for addressing the question, “How to prevent COVID-19 spread” early on in the pandemic. The question leads to an iterative process of refining the question (unbound text), searching a resource/website (blue rounded box), finding a result of interest (orange oval). Blue arrows indicate a question refinement step. Purple arrows indicate a result review step (i.e.- identifying a potential result of interest). Red arrows indicate a manual search and filter step. Green arrows indicate linked search (i.e.- manual input to a resource not needed for result).
