# Supplementary material for: Outbreak.info Research Library: A standardized, searchable platform to discover and explore COVID-19 resources and data
Source: bioRxiv. 2022 Feb 1:2022.01.20.477133. Preprint. [Version 2] doi: 10.1101/2022.01.20.477133 (PMC8820656; doi:10.1101/2022.01.20.477133)

Supplemental Figures

Supplemental Figure 1 - User-friendly API access interface which can auto-generate example queries or allow users to write and test their own queries.

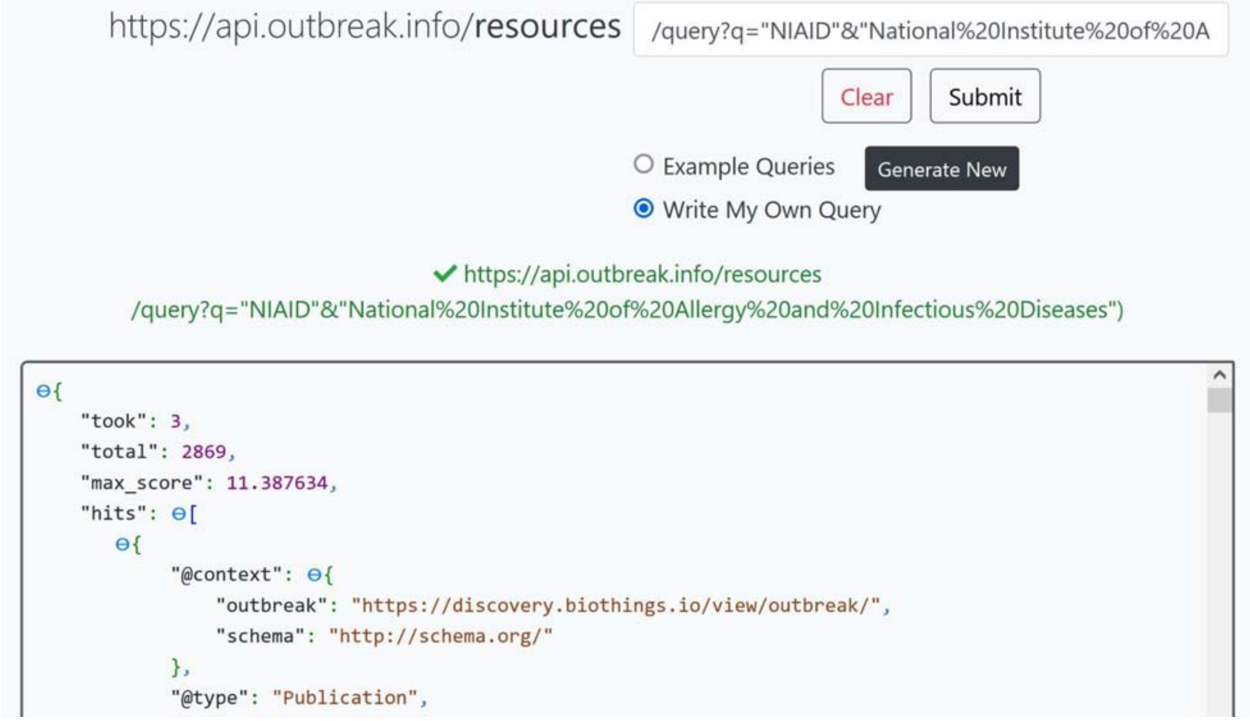

Supplement: Supplementary file 1 [file NIHPP2022.01.20.477133V2-supplement-1.pdf]
